# Petroleum Coke in the Urban Environment: A Review of Potential Health Effects

**DOI:** 10.3390/ijerph120606218

**Published:** 2015-05-29

**Authors:** Joseph A. Caruso, Kezhong Zhang, Nicholas J. Schroeck, Benjamin McCoy, Shawn P. McElmurry

**Affiliations:** 1 Institute of Environmental Health Sciences, Wayne State University, Detroit, MI 48201, USA; 2 Center for Molecular Medicine and Genetics, Wayne State University, Detroit, MI 48201, USA; E-Mail: kzhang@med.wayne.edu; 3 Transnational Environmental Law Clinic, Wayne State University, Detroit, MI 48201, USA; E-Mails: nschroeck@wayne.edu (N.J.S.); mccoyb88@gmail.com (B.M.); 4 Department of Civil & Environmental Engineering, Wayne State University, Detroit, MI 48073, USA; E-Mail: s.mcelmurry@wayne.edu

**Keywords:** petroleum coke, petcoke, urban health, environmental toxicology

## Abstract

Petroleum coke, or petcoke, is a granular coal-like industrial by-product that is separated during the refinement of heavy crude oil. Recently, the processing of material from Canadian oil sands in U.S. refineries has led to the appearance of large petcoke piles adjacent to urban communities in Detroit and Chicago. The purpose of this literature review is to assess what is known about the effects of petcoke exposure on human health. Toxicological studies in animals indicate that dermal or inhalation petcoke exposure does not lead to a significant risk for cancer development or reproductive and developmental effects. However, pulmonary inflammation was observed in long-term inhalation exposure studies. Epidemiological studies in coke oven workers have shown increased risk for cancer and chronic obstructive pulmonary diseases, but these studies are confounded by multiple industrial exposures, most notably to polycyclic aromatic hydrocarbons that are generated during petcoke production. The main threat to urban populations in the vicinity of petcoke piles is most likely fugitive dust emissions in the form of fine particulate matter. More research is required to determine whether petcoke fine particulate matter causes or exacerbates disease, either alone or in conjunction with other environmental contaminants.

## 1. Introduction

Petroleum coke (petcoke) is a granular coal-like product that is separated during the refinement of crude oil. Coking is not a new technology, as the first modern coker came online in the U.S. in the 1930s, and currently about half of U.S. petroleum refineries utilize coking technologies. However, recent upgrades to refineries in Detroit and Chicago, which allow them to process heavy crude from Canada’s oil sands projects, have sparked international attention due to the large piles of petcoke that have accumulated in urban settings. Proposals for new pipelines and increases in rail deliveries of heavy crude suggest that the production of petcoke will continue to increase in the near future. The purpose of this literature review is to assess what is known about the effects of petcoke exposure on human health.

A coker operates on the principle of thermal “cracking,” where heat is applied to break large hydrocarbon molecules into smaller fragments. Carbon is then removed as coke, leaving behind the more valuable liquid hydrocarbons. Depending on the quality of the diluted heavy crude, up to 30% (by weight) of the feeder material can be removed as solid petcoke. This green coke requires additional thermal processing, called calcining, in a rotary kiln to eliminate any residual volatile matter and increase the percentage of elemental carbon. Approximately 75% of the worldwide production of petcoke is utilized as a source of combustible fuel, whereas higher grades of calcined coke are used in steel and titanium dioxide production and the manufacture of graphite electrodes used in aluminum smelting [1]. Additional details regarding the coking process are described by Maxim *et al.* [1].

Petcoke is a carbonaceous hydrophobic black solid material (Table 1). The overwhelming majority of petcoke is a hard glassy substance that resembles coal, yet a small fraction consists of carbonaceous fibers [1]. Approximately 90% (by mass) of petcoke is composed of carbon while hydrogen, nitrogen, oxygen and sulfur constitute most of the remainder. In general, petcoke contains relatively high concentrations of silicon and trace metals which can be used to classify the source of oil [2]. Variability in petcoke composition results from differences in origin of the source material, coking temperatures and the length of coking time. About 9%–21% of green coke is composed of residual hydrocarbons, referred to as volatiles, which are removed by calcination [3]. The less volatile constituents captured within a hardened carbon matrix include polycyclic aromatic hydrocarbons (PAHs) and metals. The levels of metals present in petcoke vary depending on sources but nickel and vanadium are particularly high and often exceed 100 ppm [2]. While petroleum coke is often described as a hard glass-like substance, grinding of petcoke into smaller particles can lead to the release of volatile compounds (e.g., PAHs) and leachable metals (e.g., vanadium). 

**Table 1:** Representative composition of green petcoke [4,5].

Component	Micronized (% weight)	Trace Metals	Pellet (ppm)	PAHs	Pellet (ppm)	Micronized (ppm)
Carbon	89.8 ± 0.2	Vanadium	1748 ± 268	2-methyl naphthalene	11.5 ± 0	26 ± 0
Hydrogen	4.2 ± 0.7	Silicon	415 ± 464	Benzo[*a*]pyrene	1.8 ± 0.1	12 ± 1.4
Nitrogen	3.3 ± 0.08	Nickel	343 ± 34	Naphthalene	3.6 ± 0	11 ± 0
Oxygen	1.7 ± 0.4	Aluminum	263 ± 82	1-Methyl naphthalene	2.9 ± 0.3	11 ± 1.4
Sulfur	1.1 ± 0.06	Iron	263 ± 67	Dibenzo[*g,h,i*]perylene	1.2 ± 0.2	10.4 ± 2.3
Ash	0.2 ± 0.05			Chrysene	0.99 ± 0.16	9.7 ± 0.4
				Phenanthrene	0.66 ± 0.04	8 ± 0.3
				Benzo[*a*]anthracene	0.58 ± 0.01	7.5 ± 0.6
				Dibenzo[*a,h*]anthracene	0.50 ± 0.01	4.2 ± 0.1
				Benzo[*b*]fluoranthene	0.57 ± 0.07	3.8 ± 0.1
				Anthracene	0.14 ± 0.14	3.4 ± 0.2

## 2. Environmental Studies

### 2.1. Aquatic Organisms

Petcoke is a hardened residuum of mostly carbon, and is therefore poorly soluble in water. Nonetheless, Baker *et al.* [6] detected elevated levels of nickel in water from constructed wetlands using petcoke and consolidated tailing waste materials in Alberta, Canada. Furthermore, this study found statistically insignificant increases in nickel and vanadium levels in the green alga *Chara* *spp* [6]. In a second study based within the Alberta tar sands, Puttaswamy *et al.* [7] also reported high levels of nickel and vanadium in water leachates, this time collected from a lysimeter buried in petcoke and overlaid with soil. Toxicity testing determined that undiluted recovered water was acutely toxic to the water flea *Ceriodaphnia dubia*, and that survival of *C. dubia* correlated with the levels of vanadium and nickel in the samples [7]. In an earlier study, Pollumaa *et al.* [8] extracted contaminants from petro-chemical industrial waste materials that included fresh or aged semi-coke in Estonia. Control soil and industrial waste samples were incubated for 24 h with water (333 g/L) with shaking, and then filtered. Heavy metal levels, including nickel, were found to be low, but vanadium was not assayed. Results from toxicity testing using an alga (*Selenastrum capricornutum*), crustaceans (*Thamnocephalus platyrurus* and *Daphnia magna*), a rotifer (*Brachionus calyciflorus*), a protozoan (*Tetrahymena thermophila*) and a photobacterium (*Vibrio fischeri*) indicated that fresh semi-coke and aged polluted soils were an acute toxic hazard [8]. In a more recent report, McKee and colleagues also used water to extract contaminants in a similar fashion (24 h period with stirring), but they utilized a smaller amount of 2 mm pelleted green petroleum coke in their studies (1 g/L) [9]. Under these conditions, no toxicity was observed in an aquatic invertebrate (*D. magna*) and a fish (*Pimephales promelas*), although the growth rate of an alga (*Pseudokirchneriella subcapitata*) was inhibited by 7.1% over a 4 day observation period [9]. Levels of metals and PAHs in the water extracts were found to be either below the limit of detection or insignificant compared to control soil background concentrations. Petcoke is often stockpiled adjacent to urban waterways for shipment by merchant vessels. More studies are required to determine the risks to aquatic biomes that would result from leaching of petcoke piles after rainfall, either directly into rivers or lakes or through storm sewers, or the effect of petcoke discharge directly into waterways such as after a tanker spill.

### 2.2. Terrestrial Organisms

To study the effects of petcoke on earthworms and plants, McKee and White [9] mixed green petcoke (3.3 μm particle size) with artificial soil at 1:1000 (w/w). Under controlled conditions, no changes in appearance, behavior, or mortality was observed in adult earthworms (*Eisenia fetida*) grown in soil spiked with petcoke *versus* unadulterated test soil. Similarly, the emergence, growth and survival of corn (*Zea mays*), radish (*Raphanus sativus*) and soybean (*Glycine max*) were not significantly affected by the presence of petcoke over a 21 d period [9]. A second study compared growth of plants in either pure petcoke, or petcoke capped with 3 cm of soil in an attempt to replicate a scenario involving land reclamation after petcoke dispersal [10]. Results showed that wheat (*Triticum aestivum*) and grass (*Deschampsia caespitosa*) seeds survived when germinated in petcoke, but they exhibited significant stress symptoms such as decreases in growth rate and transpiration, and they accumulated potentially phytotoxic concentrations of vanadium and nickel [10]. Molybdenum accumulated in the grass shoots at concentrations reported to cause molybdenosis in ruminants. In urban environments, it is not known what level of fugitive dust from petcoke production, storage and transportation would be required to produce a measurable effect on local plant life. Certain plant species are known to accumulate metals at a faster rate than others, and therefore bio-remediation may be one way to mitigate the effects of petcoke airborne particulate matter (PM).

## 3. Toxicological studies relevant to human health

### 3.1. Genetic Toxicity

The mutagenicity of petcoke was analyzed by Monarca *et al.* using *Salmonella typhimurium*/microsome mutagenicity tests [11]. Petcoke and airborne particulate samples from an Italian carbon electrode factory were extracted with four organic solvents with increasing polarity: benzene, chloroform, methanol and acetone. The samples were dried, resuspended in DMSO and applied to strains TA98 or TA100 with or without metabolic activation (liver S-9 fraction). However, none of the samples demonstrated significant mutagenic activity by the criteria chosen [11]. Jongeneelen *et al.* [12] performed a similar assay but obtained different results. Petcoke samples were extracted by sonication in DMSO for 30 min and then the suspension was fed to TA98 and TA100 strains with or without metabolizing enzymes. The authors reported a concentration-dependent doubling of the number of revertants in the presence of S-9 liver extract [12]. Dalbey and co-workers [13] utilized a modified assay in which the petcoke was dissolved in cyclohexane and subsequently dissolved in DMSO. In addition, only strain TA98 is tested, and hamster S-9 liver homogenate is used. For this assay, green petcoke tested positive for mutagenicity [13]. Cumulatively, these studies suggest that green petcoke contains components that are mutagenic, however, the polycyclic aromatic hydrocarbons trapped therein would not be expected to be readily released under environmental conditions.

### 3.2. Carcinogenicity

Two studies determined the carcinogenicity of petcoke in either rats (Sprague-Dawley) or monkeys (*Cynomolgus*) using inhalation of micronized fine particles (average particle size = 3 μm). Animals were exposed for 6 h per day for 5 days per week at either 0, 10 or 30 mg/m^3^. No excess tumor formation was found at the end of the 2 year observation period [14,15]. To determine if a dermal route of exposure was carcinogenic in mammals, petcoke was micronized, suspended in mineral oil, and painted on the skin of C3H/HeJ mice 3 times per week for 2 years. Results from this study were negative for tumor formation [16]. Therefore, *in vivo* carcinogenicity assays indicate that petcoke exposure does not lead to a higher incidence of cancer.

### 3.3. Reproductive and Developmental Toxicity

A reproductive/developmental toxicity screening test (OECD 421) was performed using green petcoke [17]. Sprague-Dawley rats (12 male and 12 female) were exposed to 0, 30, 100, or 300 mg/m^3^ micronized petcoke via nose inhalation for 6 h per day for 14 days prior to mating. Exposure of males and females was continued during the mating period and gestation. Standard reproductive indices in parents and developmental characteristics in pups were evaluated, and no toxic effects were reported in the parents or the offspring [17]. McKee *et al.* performed a similar OECD 421 screening using the same dosing regimen. They reported that 3 of 12 female rats within the 300 mg/m^3^ group did not become pregnant, although two had copulatory plugs which indicated that mating had occurred. An additional female became pregnant but not give live birth [9]. All offspring appeared normal and bore no significant *in utero* developmental defects. The authors concluded that green petcoke demonstrated low reproductive and developmental toxicity.

### 3.4. Repeated-Dose Toxicity

In the 2 year inhalation studies discussed in Section 3.2, the animals were examined for signs of toxicity. In addition to gross morphology, 31 tissues from 10 female and male rats were microscopically examined at 3, 6, 12, and 18 months, as well as all remaining animals at 24 months. For monkeys, the same 31 tissues were examined only after 24 months exposure. There were no reported petcoke exposure-related effects on body weight or mortality in either rats or monkeys. Of all the tissues examined, dose-related effects were only observed in the trachea and lungs. In both rat and monkey there were reported increases in the weights of the lung and trachea which was associated with accumulation of neutrophils and leukocytes and a decrease in lymphocytes. This effect was accompanied by pigment accumulation in the lung and associated lymph nodes. For rats, lung histo-pathological changes included bronchiolization (adenomatous hyperplasia), sclerosis, squamous alveolar metaplasia (keratin cysts), and pulmonary interstitial inflammatory responses with focal fibrosis. The severity of these effects correlated with the duration and concentration of petcoke exposure, and was described as non-reversible. Monkey lung tissue presented lymph node pigment accumulation that resulted from phagocytosis via pulmonary macrophages, but other changes observed in rats were not detected. 

Although the number of reports and population sizes were small, the overall conclusion from these studies is that petcoke is not carcinogenic via inhalation or dermal exposure, there is low risk of developmental toxicity but some risk of reproductive effects, and that the primary health effect via inhalation is pulmonary inflammation.

## 4. Biomonitoring of Human Exposures

Assessing the hazard associated with an environment exposure requires delineating the dispersion of the toxicant in an attempt to estimate dose. For example, an air monitoring station or water sampling from a river along with geographic modeling can alert regulators of the potential impact of known carcinogens. A more detailed assessment can be made when human biomonitoring is included since variabilities in exposure can result from inter-individual differences in movements (e.g., indoor *vs.* outdoor, types of outdoor activities, time spent at home compared to work), diet, genetics, as well as other sources. Biomonitoring in humans can encompass different routes of exposure, including inhalation, ingestion and dermal uptake, and takes into account variation in absorption, metabolism and elimination by the body.

One of the greatest limitations in developing valid dose-response extrapolation models in humans is the lack of quantitative data on the relationship between external exposure and an effective dose to specific target molecules, cells or organs. The development of molecular and biochemical methods to measure internal exposure (e.g., urinary metabolites) and biological effective doses (e.g., DNA and protein adducts) has facilitated human exposure-dosimetry biomarker studies. The biomarker and epidemiological studies to date have been limited to coke oven workers.

### 4.1. 1-Hydroxypyrene

1-Hydroxypyrene (1-HP) is considered to be the best biological biomarker for measuring exposure to polycyclic aromatic hydrocarbons (PAHs). Pyrene is found in high percentages in all mixtures of PAHs, including the PAHs found in petroleum coke [12]. In humans, about 90% of pyrene is converted to 1-HP, which is excreted in the urine as glucuronide and/or sulphate conjugates, with an average urinary half-life of 18 h [18]. 1-HP can be measured in extracted urine samples by HPLC. Environmental studies have found associations between PAH exposures and 1-HP levels, for example, urinary excretion of 1-HP has been correlated with PM less than 10 micron (PM_10_) in urban environments surrounding a steel mill [19]. In a review of environmental and occupational exposure studies, Hansen *et al.* found that coke-oven workers had the highest levels of urinary 1-HP, particularly in work places from Taiwan and China [20].

### 4.2. DNA Adducts

^32^P-Postlabeling analysis is a method for detection of bound environmental contaminants to DNA. The procedure involves five main steps: enzymatic digestion of a DNA sample; enrichment of the adducts; radiolabeling of the adduct; chromatographic separation of labeled adducts; and detection/quantitation [21]. The sensitivity of this technique allows the evaluation of DNA adducts at sufficiently minute levels that the question of low-dose extrapolation in humans with respect to exposure estimates can be tested. Lewtas *et al.* [22] utilized this methodology to determine if there was a correlation between personal exposure (subjects carried a portable air sampler) and DNA adducts in purified white blood cells. For these studies, Czech petroleum coke oven workers in 10 job categories were compared to a control population drawn from the surrounding area. Results showed that at low to moderate environmental exposure to PAHs, DNA adducts levels significantly correlated with exposure. However, this relationship became non-linear at the higher occupational levels found in coke oven workers. It was postulated that saturation of metabolic activation enzymes and induction of detoxification processes could be contributing factors to explain this effect [22]. In a similar study, Farmer *et al.* [23] found a positive correlation between estimated exposure of coke oven workers and PAH-DNA adducts levels. For these studies, both ^32^P-postlabelling and a radioimmunoassay against BPDE-DNA adducts levels were used.

These studies highlight advancements in the sensitivity in detection of human PAH exposure, however, it cannot be determined whether the PAHs originated from petcoke particulates or from other sources during the coking process.

## 5. Epidemiology

Epidemiological studies are complicated by the variable chemical composition of petcoke and the often co-location of petcoke with other industrial sources of pollution. The two most prominent trace metals typically found in petcoke, nickel and vanadium, are common to other petroleum-based emission sources. As a result, the combustion of fossil fuels are responsible for nearly all of the anthropogenic sources of nickel (90%) and vanadium (100%) [24]. While the emission of many trace metals have declined in recent years due to increased environmental awareness and regulation, the amount of nickel and vanadium have steadily increased with oil consumption. Mixtures of PAHs are also ubiquitous due to an array of combustion sources (e.g., power plants, vehicle exhausts) as well as other non-occupational sources including tobacco smoke and diet (e.g., via grilling). Therefore, comparison of occupationally-exposed populations to reference populations may be biased to an unknown extent.

### 5.1. Cancer

During the coking process, volatile substances including PAHs are burnt off. Coke oven workers are most susceptible to cancer when they are located at the top of the oven compared to the side [25]. In large cohort studies (n > 15,000) in North America [26,27] and China [28] there was up to a 2- to 4.4-fold increase in the risk of developing cancers of the lung and bronchus (Table 2, based on data from [29]). Similar results were found in a smaller study from France (n = 536, [30]), but other studies (n < 8000) in the UK [31,32,33], Japan [34] and The Netherlands [35] reported relative risk values in the range of 0.8–1.4. For prostate cancer, relative risk was as high as 1.93 (CI = 1.11–3.21) for workers in USA and Canada [26]. No evidence of risk for other tumor types, including bladder cancer, was reported.

**Table 2:** Cohort studies of lung cancer in coke production workers from various countries.

Country	n	RR	CI	Notes
USA & Canada	15,818	2.0	1.6–2.3	Coke oven workers
Canada	25,292	2.17 *	0.87–4.48	Highest exposure tertile
China	21,995	4.4	3.3–5.8	Subset of coking dept. workers
France	536	2.5	1.1–5.0	Subset of near ovens workers
UK	610	0.8	0.4–1.6	Coke oven workers
UK	2842	1.2	1.0–1.4	Subset of coke plant workers
UK	8000	1.4	0.8–2.3	Coke oven workers
Japan	2178	1.3	0.7–2.1	Coke oven workers
Netherlands	5639	1.3	1.0–1.7	Subset of coke oven workers

Notes: * Standard incidence ratio, n = study population, RR = relative risk, CI = confidence interval.

### 5.2. Chronic Obstructive Pulmonary Diseases

The main symptom of chronic obstructive pulmonary disease (COPD) is the progressive decline in the ability to breathe, and includes emphysema and chronic bronchitis. Cigarette smoking is the major risk factor of COPD, but 20% of cases may be attributable to occupational exposures [36]. After adjusting for cigarette smoking and other risk factors, Hu and colleagues found a 5.8-fold increase in risk for developing COPD in coke oven workers with the highest levels of exposure to emissions [37]. Furthermore, up to a 2.2-fold increase in relative risk of death from non-malignant respiratory diseases was reported in a large cohort of US coke workers [25]. While exposure to PM is associated with acute inflammatory response, the bioavailability of contaminants present in the PM is critical in defining an associated hazard. Costa and Dreher [38] found the dose of bioavailable transition metals present in PM (residual oil fly ash), not instilled PM mass, was the primary determinant of the acute inflammatory response in rats. While this work was not specifically on petcoke, it is reasonable to assume that human risk to cardiopulmonary injury is similarly associated with the bioavailability of metals present in petcoke.

## 6. Exposure to Petcoke Fine Particulates in Urban Environments

Based on the glassy nature of petcoke, it is unlikely that metals and organic material from undisturbed petcoke will leach into surface material and groundwater. For the same reasons, risk associated with dermal exposure is improbable. Because petcoke is an industrial byproduct, potential for ingestion is unlikely. However, incidental ingestion to fugitive dust and fibers generated by the processing of petcoke can occur. Therefore, the primary pathway for human exposure to petcoke is via inhalation of petcoke dust generated during handling, storage and transport.

The potential generation of fugitive dust from petcoke piles is a concern for communities in Chicago, Detroit, and elsewhere petcoke storage piles and processing facilities have been sited proximate to residential areas. An important difference between petcoke and coal is the silt content: 21.2% for petcoke compared to 4.6% for coal in one report [39].Therefore, of these two stockpiled fuel sources, the risk of fine PM blowing into surrounding neighborhoods is greater for petcoke. It has been estimated that about 100 tons of petcoke fugitive dust are released into the atmosphere per year in the US [39].

While it is difficult to definitely identify petcoke dust, primarily because these piles are in or near facilities that process petrochemicals with similar elemental characteristics, some groups have described airborne transport of fugitive dust from petcoke. The enrichment of nickel and vanadium in petroleum and petcoke has led to the use of these elements to apportion sources of PM (e.g., Pandolfi *et al.* [40]). Most directly, Moreno *et al.* [41] detected airborne PM with high vanadium/nickel ratios (>4) nearby petcoke emission sources in Spain. More research is needed to identify definitive signatures of petcoke in urban PM and quantify potential fugitive emissions from petcoke production and handling.

## 7. Discussion

The sudden emergence of large piles of petcoke along waterways adjacent to Detroit and Chicago led to apprehension among local residents about the possibility of deleterious health effects that could result from exposure. Inhalation is the most prevalent concern, as black dust was observed to be blown off the piles under extreme weather conditions, and was found to accumulate on residential properties in the vicinity of stockpiled petcoke. Airborne petcoke has the potential to exacerbate pre-existing lung ailments, or may have additive or synergistic effects with other environmental toxins. Southwest Detroit, where petcoke was stored in open piles, contains several industrial sources of pollution and major transportation networks and has higher rates of lung and bronchus cancers compared to the rest of the city [42]. Furthermore, the incidence of adult asthma is 50% higher in Detroit than in the rest of the Michigan, and the asthma death rates are double [43]. Chicago, a city currently impacted by the open-air storage of petcoke, similarly suffers from higher than average rates of asthma when compared to the rest of the state of Illinois [44]. The neighborhood where the petcoke is currently stored, South Deering, has particularly high rates of asthma, as do many of the surrounding neighborhoods of Southeast Chicago [44].

The current knowledge of petcoke toxicity may not adequately address the risk of urban exposure. Mutagenicity studies indicate it is not likely to be carcinogenic under normal exposure conditions. However, the potential of petcoke to exacerbate lung disease such as asthma and COPD is not currently known. Studies on human petcoke exposure have been limited to coke oven workers, but this is not indicative of a residential exposure due to such factors as differences in coking operations in different industries and in various countries, and exposure to multiple toxins within the refinery. The most prominent threat to urban environmental health is most likely as a fine PM. Ambient PM in fine and ultrafine ranges (aerodynamic diameter < 2.5 μm, PM_2.5_) is strongly associated with the pathogenesis of air pollution-associated systemic diseases [45,46]. Epidemiological and animal model studies have linked air pollutant PM_2.5_, primarily derived from stationary and traffic-related combustion sources, to the increase of mortality and morbidity associated with asthma, lung cancer, cardiovascular disease, metabolic disease, and birth defects [47,48,49,50,51]. Most recent studies suggest that long-term exposure to PM_2.5_ above the current US EPA standards are associated with neurodegeneration and increased risk of Alzheimer’s disease [52,53]. PM pollution is estimated to cause over 20,000 deaths per year in the United States [54]. The detrimental effects of PM pollution on human health in developing countries, such as China, India, and Africa, were significantly underestimated. In the US, Detroit is one of the top 25 US cities most polluted by year-round particle pollution according to the American Lung Association annual reports. Traffic-related PM_2.5_ is a complex mixture of particles and gases from gasoline and diesel engines, together with dust from wear of road surfaces, tires, and brakes [55,56]. Airborne PM_2.5_ shows an incremental capacity to penetrate to the distal airway units and potentially the systemic circulation [57,58]. Recent studies have indicated that traffic-related PM_2.5_ promotes systemic disease through: exaggerating systemic inflammation [51,59], enhancing oxidative damage [60,61,62], and causing endoplasmic reticulum stress [62,63] in a variety of tissues or organs.

## 8. Conclusions 

Basic toxicological research suggests that the main threat to human health from petcoke is aggravation of the respiratory system. However, little is known about the effects of petcoke as a fine PM on exposed urban populations. To assist with defining environmental exposure, chemical characteristics of petcoke particles or fibers, and a more rigorous quantification of fugitive dust emissions from storage piles, are required. Finally, in order to fully understand the potential risk to community health, more research is required to determine whether petcoke fine PM causes or exacerbates disease, either alone or in conjunction with other environmental contaminants.
